# Supplementary material for: Controlled noise: evidence of epigenetic regulation of single-cell expression variability
Source: Bioinformatics. 2024 Jul 17;40(7):btae457. doi: 10.1093/bioinformatics/btae457 (PMC11283284; doi:10.1093/bioinformatics/btae457)
Supplement: btae457_Supplementary_Data [file btae457_supplementary_data.zip › OP-CBIO240465_PECorr_AttachmentsFolder_sub_manuscript[AU].pdf]

Supplementary Material for “Controlled noise: evidence of epigenetic regulation of single-cell expression variability”

Yan Zhong[1,*], Siwei Cui[1], Yongjian Yang[2], James J. Cai[2,3,4,*]

1 School of Statistics, KLATASDS-MOE, East China Normal University, Shanghai, 200062, China
2 Department of Electrical and Computer Engineering, Texas A&M University, College Station, TX 77843, USA
3 Department of Veterinary Integrative Biosciences, Texas A&M University, College Station, TX 77843, USA
4 Interdisciplinary Program of Genetics, Texas A&M University, College Station, TX 77843, USA

*Corresponding Authors: Yan Zhong, yzhong@fem.ecnu.edu.cn; James J. Cai, jcai@tamu.edu

This supplementary file includes the figures for two additional datasets and the MNN structure. **Figures S1-S6** are for the data of HSPCs from Donor #32606 at Day 2, and **Figures S7-S12** are for the data of neurons. Since the number of cells and genes for neuron data are relatively small, we only select a group of 2000 homogeneous cells and consider at most 1000 HVGs in the study of neurons. **Figure S13** shows the specific structure of the MNN model.

S1. HSPCs from Donor #32606 at Day 2

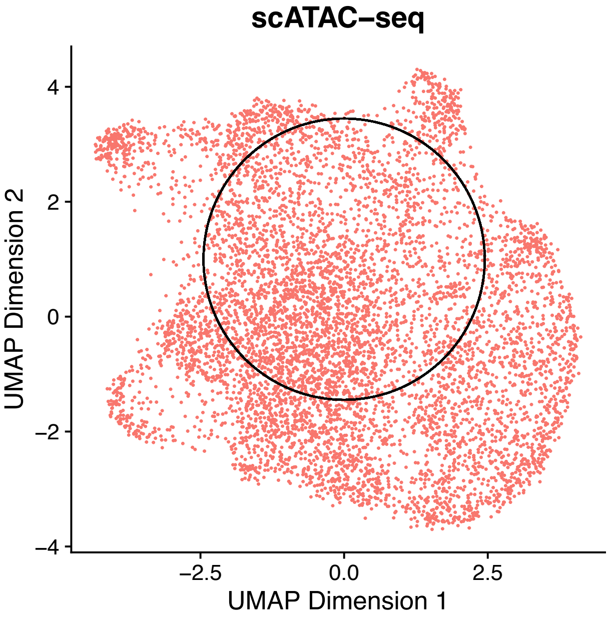

Figure S1. A homogeneous population of 3000 cells was selected for analysis of HSPCs from Donor #32606. The Signac R package was used to produce a 2-dimensional non-linear representation of the scATAC-seq data. A core cell was chosen, and the 3000 cells nearest to the core cell (including itself) were selected as a group of homogeneous cells. The selected cells are surrounded by the black circle.

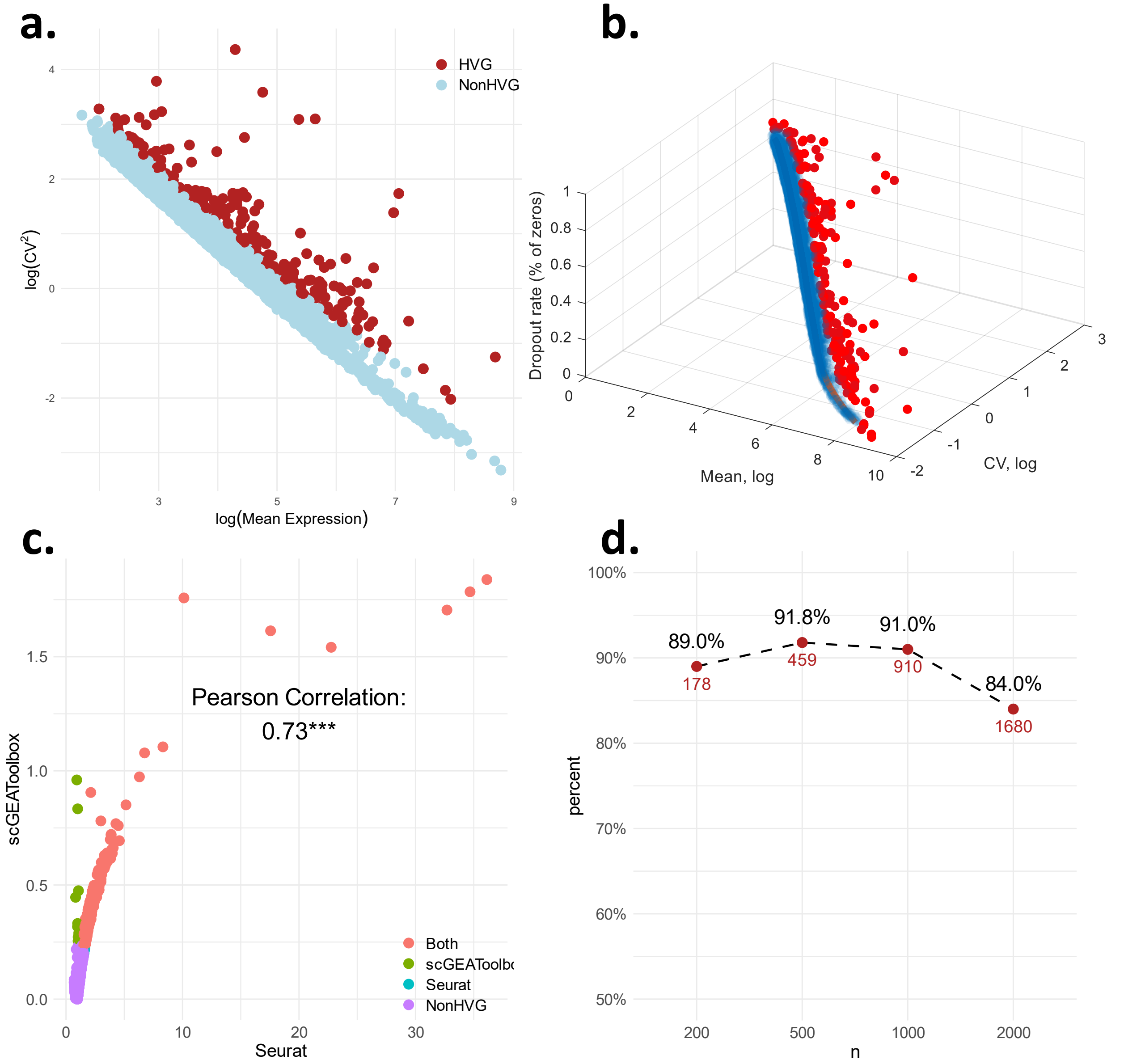

Figure S2. Results of two HVG detection methods for HSPCs from Donor #32606. (a) The 2D scatterplot of genes with $\log(\text{Mean Expression})$ as the x axis and $\log(\text{CV}^2)$ as the y axis. Top $K = 200$ genes with relatively high $\log(\text{CV}^2)$ corresponding to $\log(\text{Mean Expression})$ are identified as HVGs by Vst method in Seurat and are highlighted. (b) Splinefit method in scGEAToolbox draws the 3D scatterplot of genes in $(\log(\text{Mean Expression}), \log(\text{CV}), \text{Dropout rate})$ and fits a 3D curve. The K = 200 genes farthest from the curve are marked as the HVGs and are highlighted. (c) The scatterplot of the scEV levels of genes for two HVG detection methods. The Pearson correlation is 0.73 with p value <0.001. 178 genes simultaneously denoted as HVGs by both methods are highlighted. (d) The number and ratio of overlapped genes between two methods when selecting different numbers of HVGs (K = 200, 500, 1000, 2000).

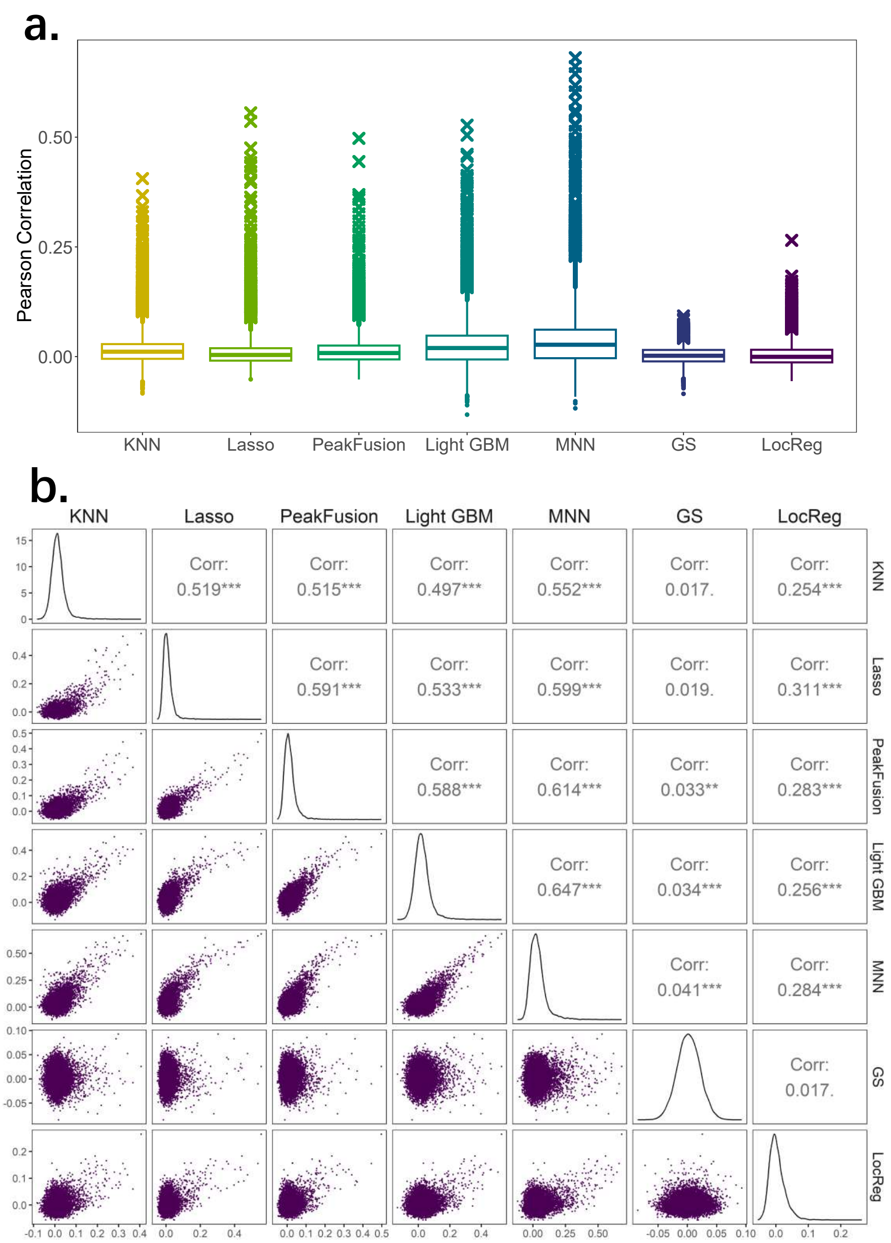

Figure S3. Results from seven prediction methods applied to the paired scATAC-seq and scRNA-seq data of HSPCs from Donor #32606. All the results represent the average results of five training-test sample splits. (**a**) Boxplot depicting Pearson correlations between true and estimated scRNA-seq levels for all genes across seven predictive models. The upper outliers in each boxplot signify genes that were well predicted with high Pearson correlations. In each model, the top 200 genes with the highest Pearson correlation coefficients are marked with a cross. (**b**) Scatter plots illustrating Pearson correlation for each pair of the seven models, with each point representing a gene.

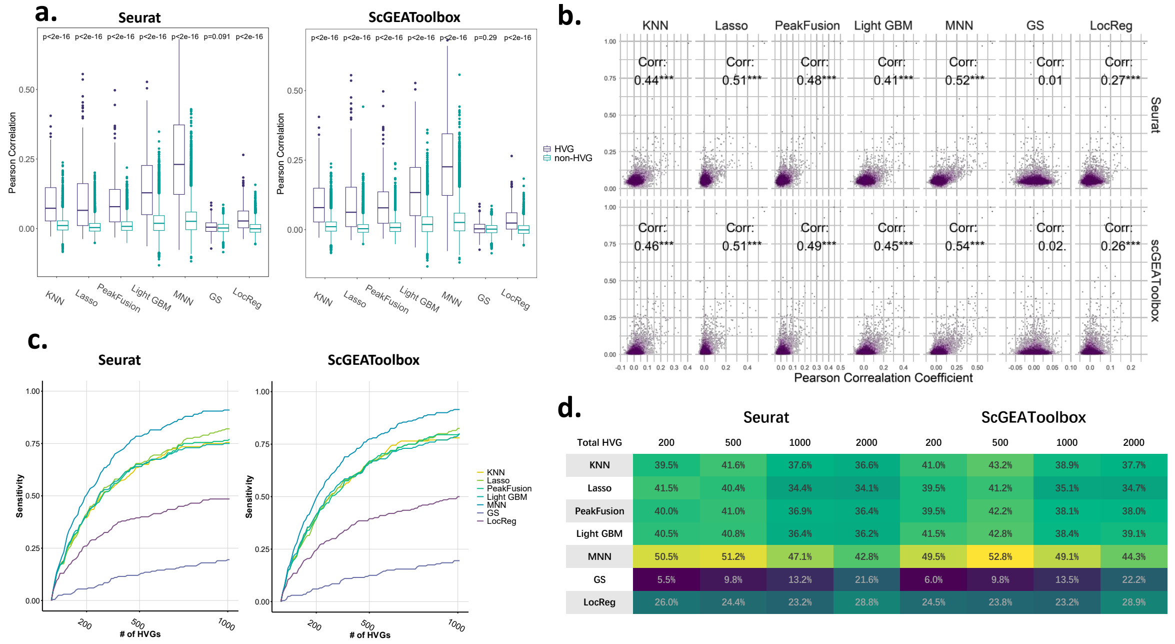

Figure S4. Prediction performance for HVGs across diverse models for HSPCs from Donor #32606. (**a**) Identification of 200 HVGs using each HVG detection method, followed by boxplots illustrating the Pearson correlation for HVGs and non-HVGs within each predictive model. The Wilcoxon test was used to compare the medians of the two gene sets. (**b**) Scatterplots presenting genes with the x-axis indicating Pearson correlation and the y-axis representing scEV levels. Each column corresponds to a predictive model, and each row corresponds to an HVG detection method. Spearman correlation was calculated and tested for each scatterplot. (**c**) The percentage of the top 200 well-predicted genes that were denoted as HVGs as the number of detected HVGs increased. (**d**) Evaluation of the overlap between the *K* well-predicted genes and the *K* detected HVGs for each pair of predictive models and HVG detection methods (K = 200, 500, 1000, 2000). Fisher's exact test was used to assess the independence between well-predicted genes and HVGs for each method pair. All pairs demonstrated significant nonindependence, with a p value < 0.001, except for (GS, Seurat) and (GS, scGEAToolbox).

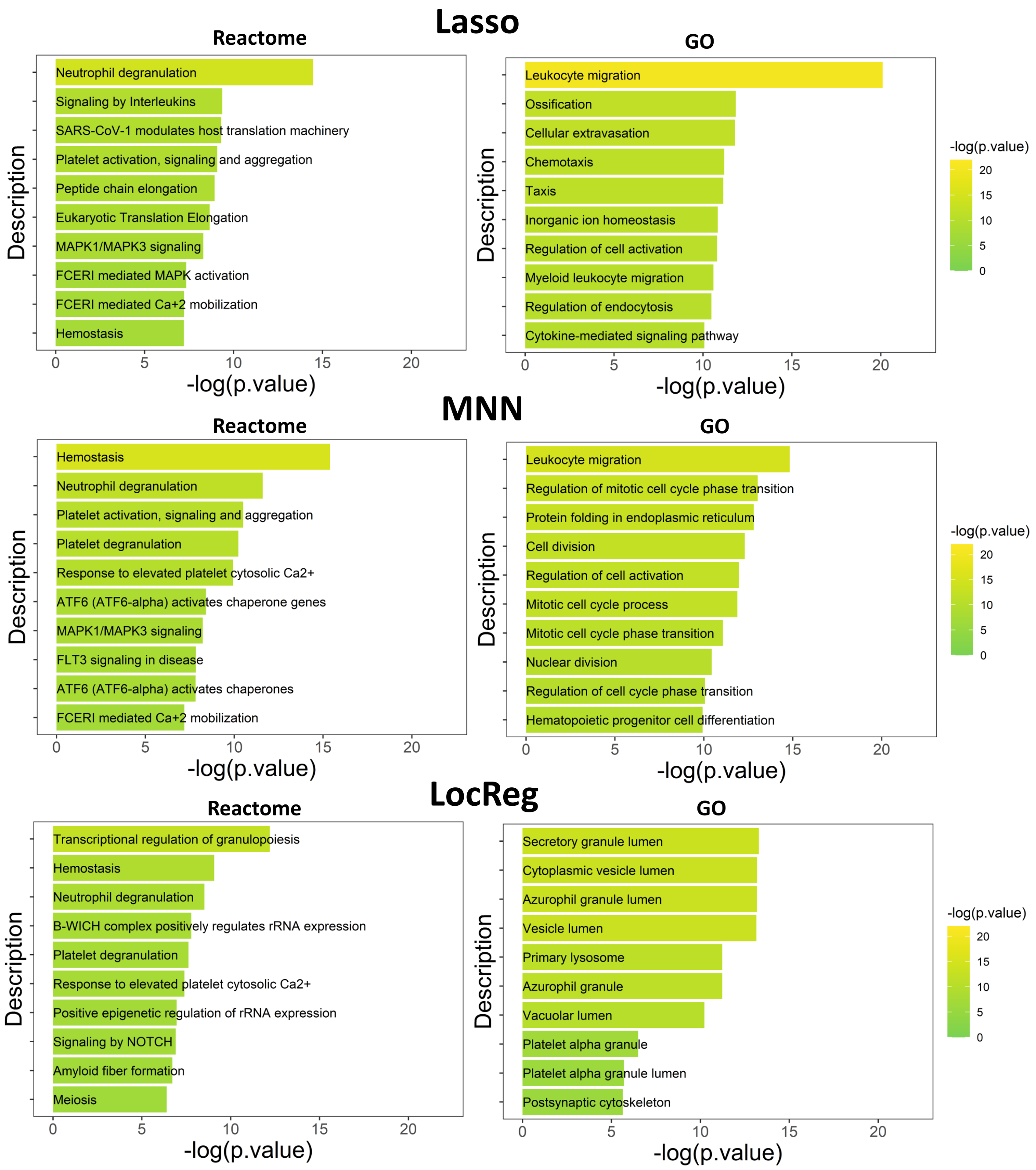

Figure S5. Lists of top 10 significantly enriched pathways in top 200 well-predicted genes according to the Lasso, MNN, and LocReg models for HSPCs from Donor #32606. Pathways that contain more than 800 genes associated with general functions are not shown.

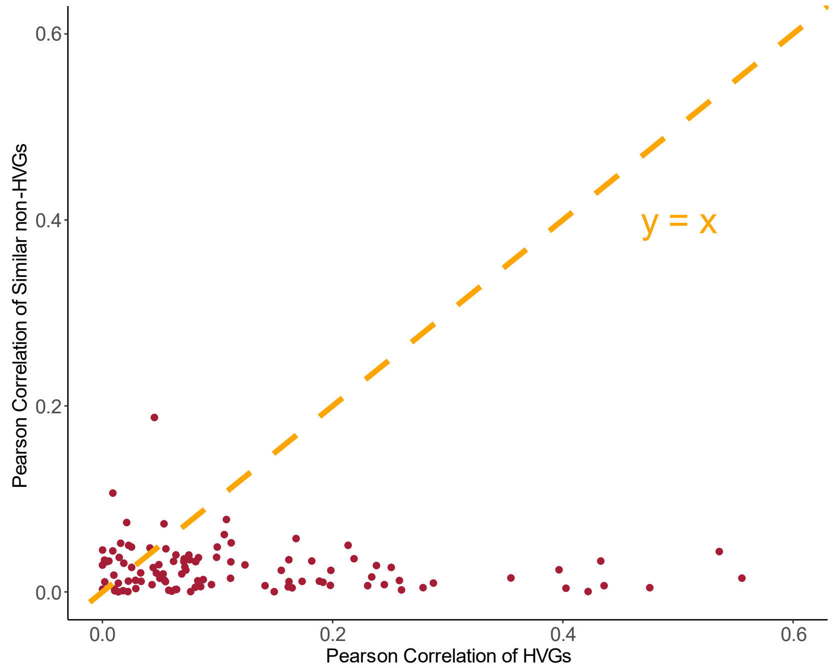

Figure S6. Prediction performance of HVGs and non-HVGs with controlled mean expression level for HSPCs from Donor #32606. For each of K = 200 HVGs by Seurat, the non-HVG with the closest mean expression level is used as a compared gene with mean expression level controlled. The scatterplot of the average Pearson correlation of each HVG and its compared non-HVG shows that HVG have much better prediction performance than its compared non-HVG.

S2. Neurons

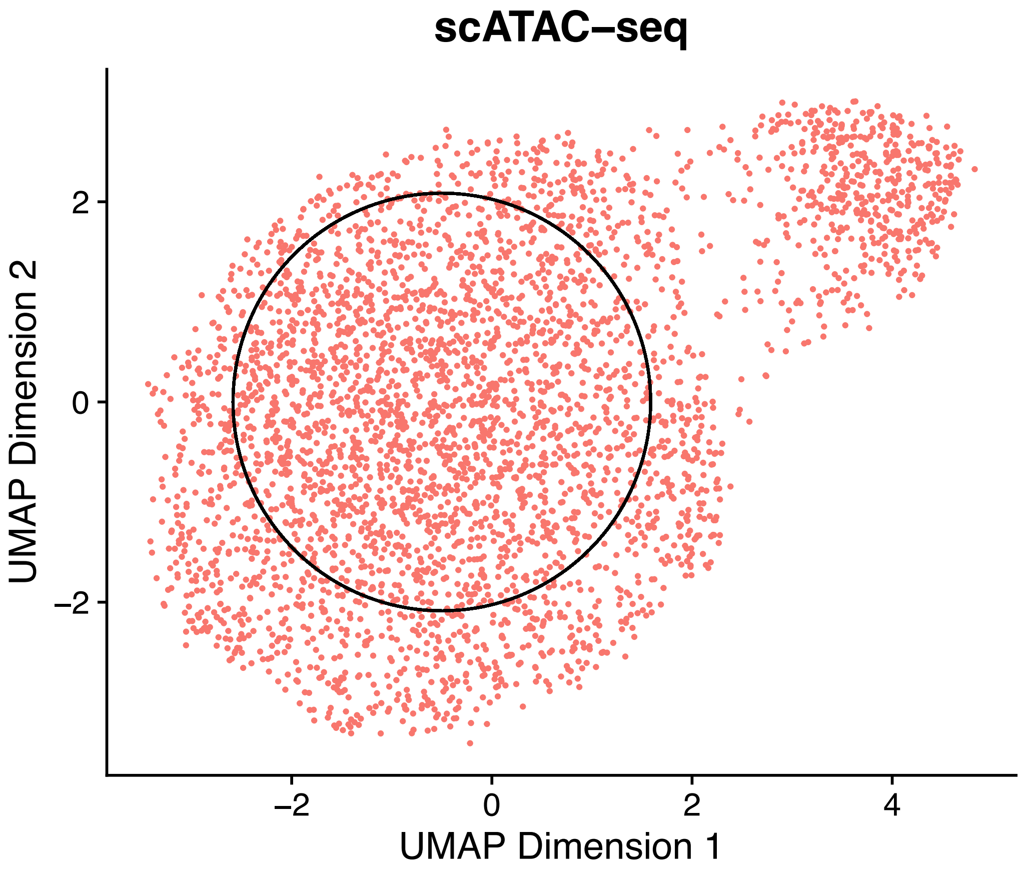

Figure S7. A homogeneous population of 2000 cells was selected for analysis of neurons. The Signac R package was used to produce a 2-dimensional non-linear representation of the scATAC-seq data. Since the total number of neurons is only 3784, we selected 2000 cells. A core cell was chosen, and the 2000 cells nearest to the core cell (including itself) were selected as a group of homogeneous cells. The selected cells are surrounded by the black circle.

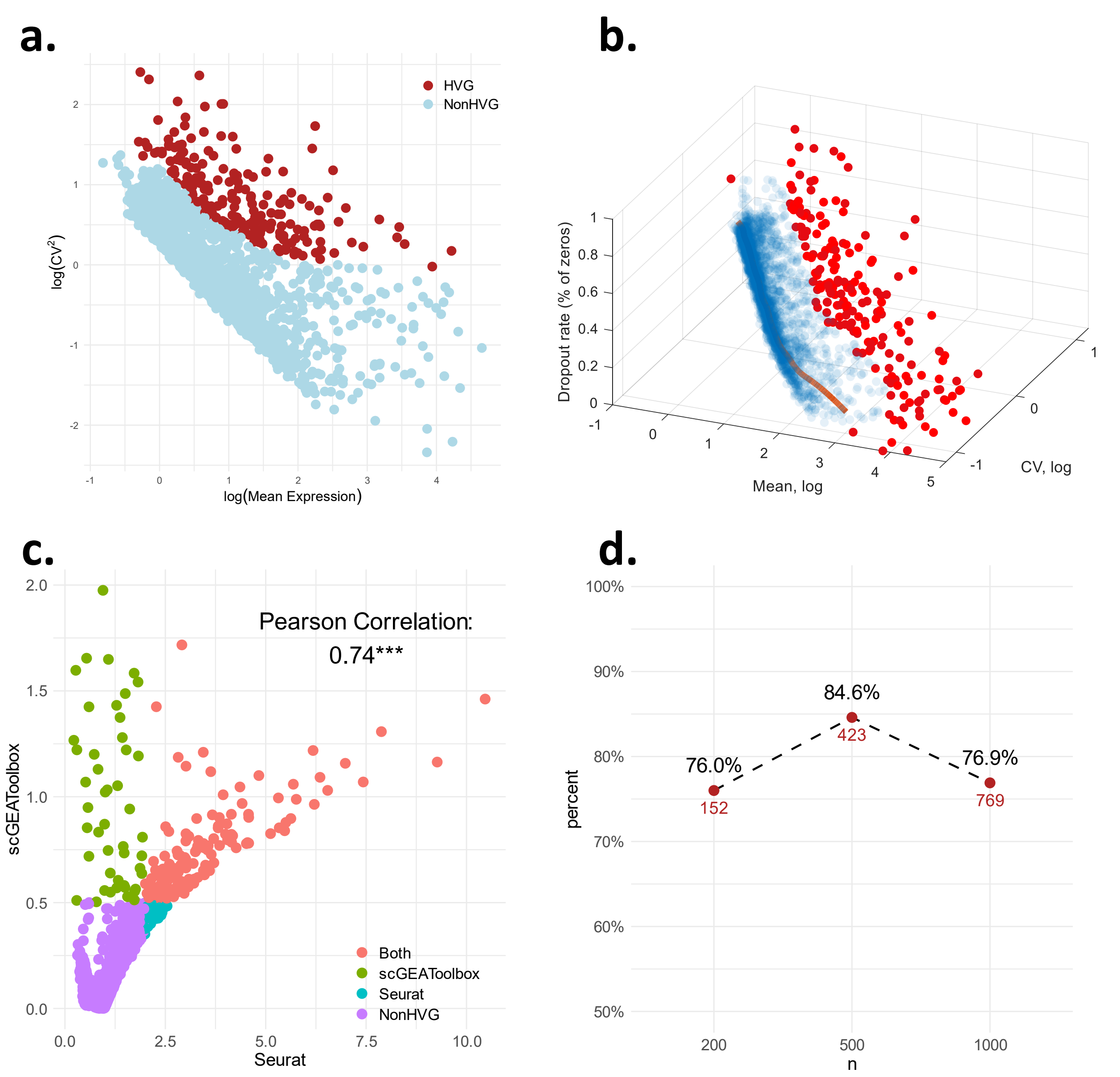

Figure S8. Results of two HVG detection methods for neurons. (a) The 2D scatterplot of genes with $\log(\text{Mean Expression})$ as the x axis and $\log(\text{CV}^2)$ as the y axis. Top $K = 200$ genes with relatively high $\log(\text{CV}^2)$ corresponding to $\log(\text{Mean Expression})$ are identified as HVGs by Vst method in Seurat and are highlighted. (b) Splinefit method in scGEAToolbox draws the 3D scatterplot of genes in $(\log(\text{Mean Expression}), \log(\text{CV}), \text{Dropout rate})$ and fits a 3D curve. The K = 200 genes farthest from the curve are marked as the HVGs and are highlighted. (c) The scatterplot of the scEV levels of genes for two HVG detection methods. The Pearson correlation is 0.74 with p value <0.001. 152 genes simultaneously denoted as HVGs by both methods are highlighted (d) The number and ratio of overlapped genes between two methods when selecting different numbers of HVGs (K = 200, 500, 1000).

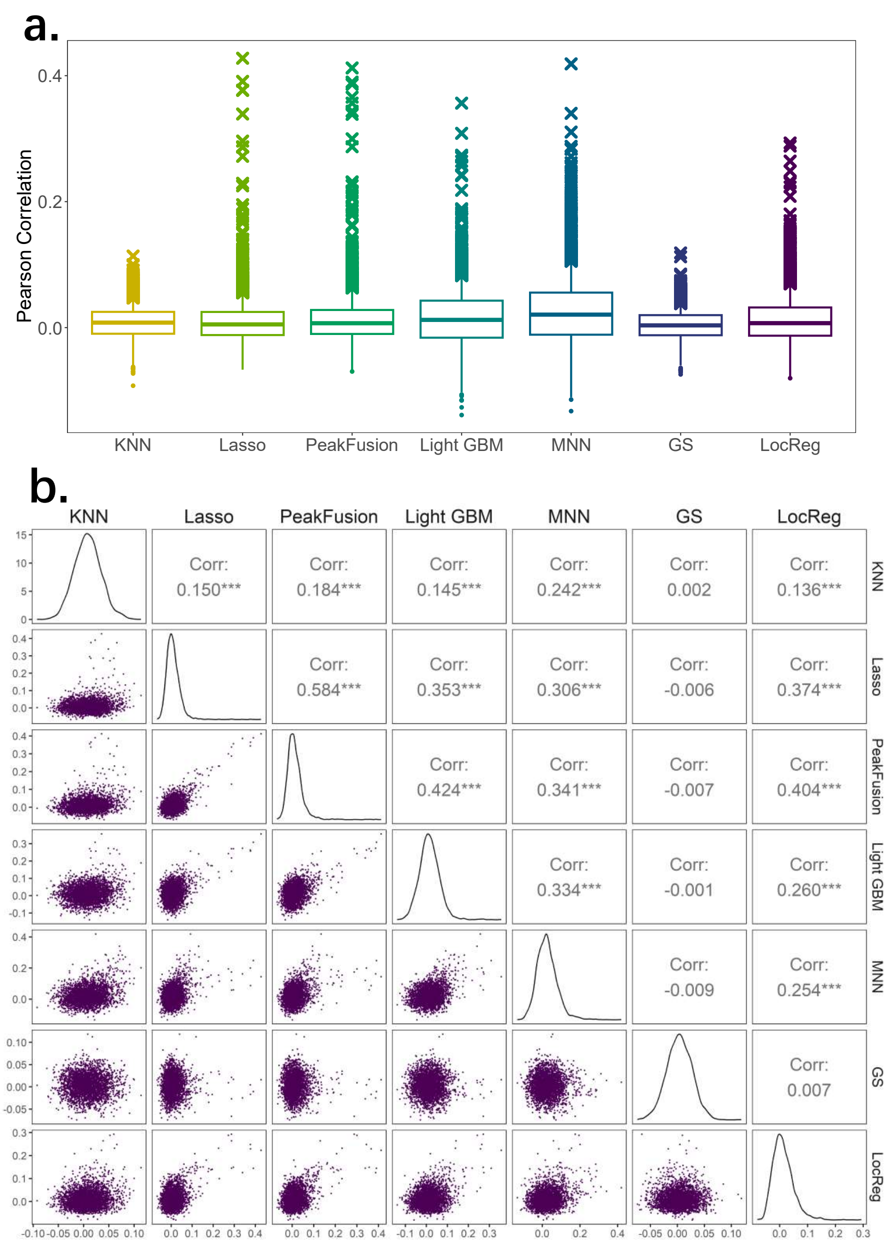

Figure S9. Results from seven prediction methods applied to the paired scATAC-seq and scRNA-seq data of neurons. All the results represent the average results of five training-test sample splits. (**a**) Boxplot depicting Pearson correlations between true and estimated scRNA-seq levels for all genes across seven predictive models. The upper outliers in each boxplot signify genes that were well predicted with high Pearson correlations. In each model, the top 200 genes with the highest Pearson correlation coefficients are marked with a cross. (**b**) Scatter plots illustrating Pearson correlation for each pair of the seven models, with each point representing a gene.

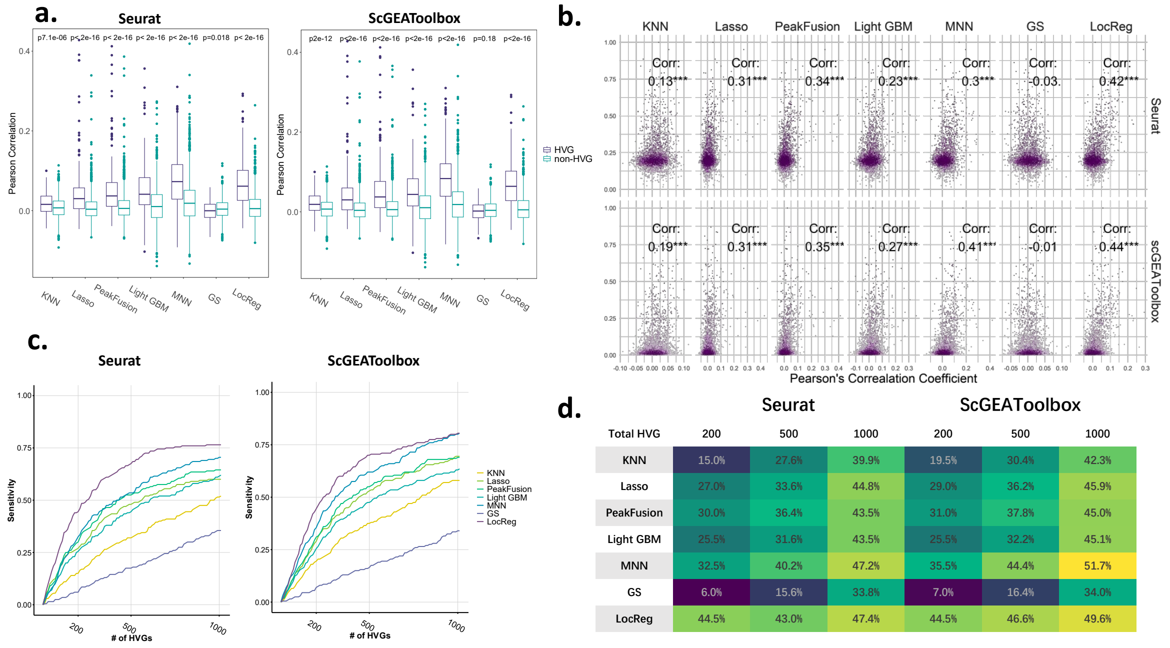

Total HVG	200	500	1000	200	500	1000
KNN	15.0%	27.6%	39.9%	19.5%	30.4%	42.3%
Lasso	27.0%	33.6%	44.8%	29.0%	36.2%	45.9%
PeakFusion	30.0%	36.4%	43.5%	31.0%	37.8%	45.0%
Light GBM	25.5%	31.6%	43.5%	25.5%	32.2%	45.1%
MNN	32.5%	40.2%	47.2%	35.5%	44.4%	51.7%
GS	6.0%	15.6%	33.8%	7.0%	16.4%	34.0%
LocReg	44.5%	43.0%	47.4%	44.5%	46.6%	49.6%

Figure S10. Prediction performance for HVGs across diverse models for neurons. (**a**) Identification of 200 HVGs using each HVG detection method, followed by boxplots illustrating the Pearson correlation for HVGs and non-HVGs within each predictive model. The Wilcoxon test was used to compare the medians of the two gene sets. (**b**) Scatterplots presenting genes with the x-axis indicating Pearson correlation and the y-axis representing scEV levels. Each column corresponds to a predictive model, and each row corresponds to an HVG detection method. Spearman correlation was calculated and tested for each scatterplot. (**c**) The percentage of the top 200 well-predicted genes that were denoted as HVGs as the number of detected HVGs increased. (**d**) Evaluation of the overlap between the *K* well-predicted genes and the *K* detected HVGs for each pair of predictive models and HVG detection methods (*K* = 200, 500, 1000). Fisher's exact test was used to assess the independence between well-predicted genes and HVGs for each method pair. All pairs demonstrated significant nonindependence, with a p value < 0.001, except for (GS, Seurat) and (GS, scGEAToolbox). Different from HSPCs, well-predicted genes by LocReg have the highest overlap with HVGs. This implies that the scEV of genes in neurons may be more controlled by peaks around them.

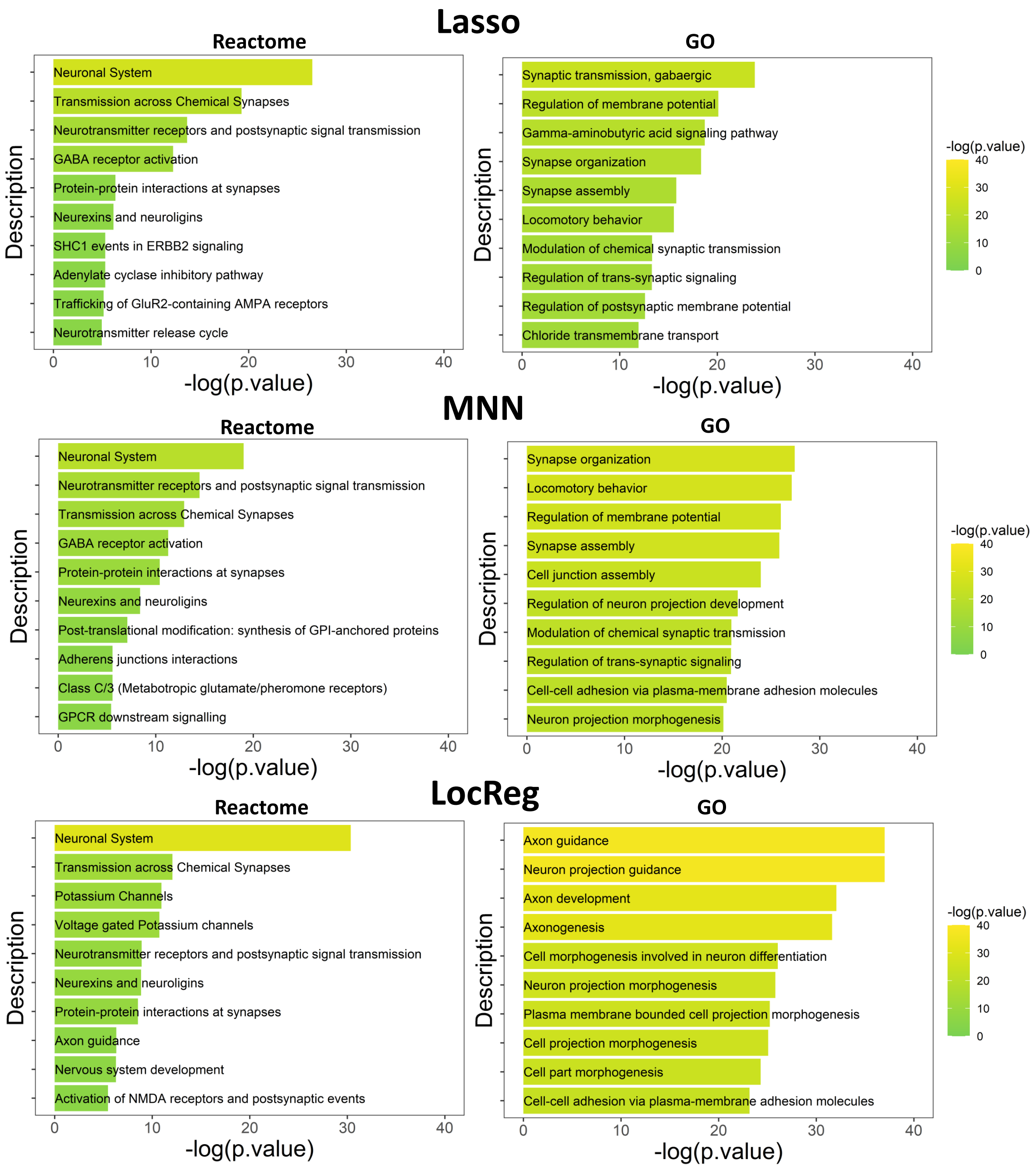

Figure S11. Lists of top 10 significantly enriched pathways in top 200 well-predicted genes according to the Lasso, MNN, and LocReg models for neurons. Pathways that contain more than 800 genes associated with general functions are not shown.

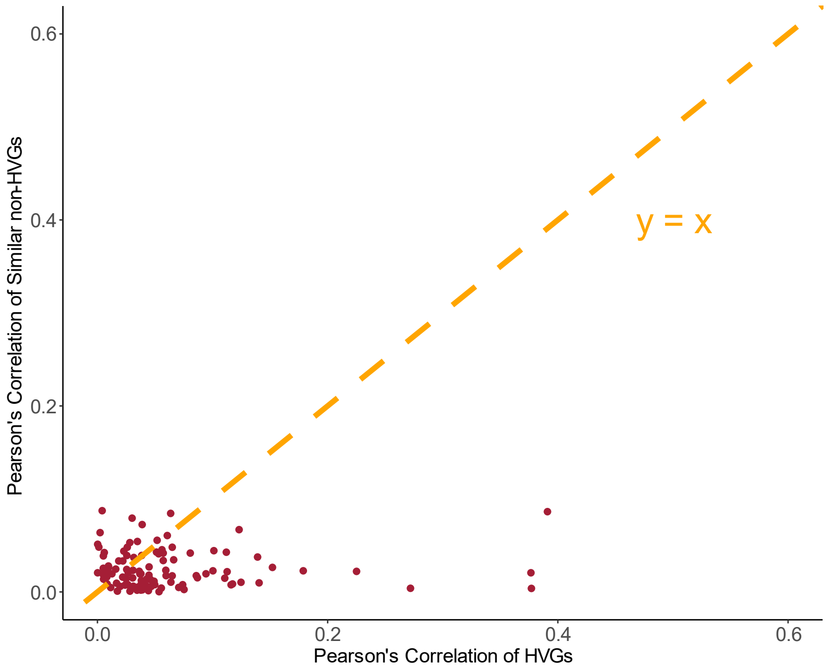

Figure S12. Prediction performance of HVGs and non-HVGs with controlled mean expression level for neurons. For each of $K = 200$ HVGs by Seurat, the non-HVG with the closest mean expression level is used as a compared gene with mean expression level controlled. The scatterplot of the average Pearson correlation of each HVG and its compared non-HVG shows that HVG have much better prediction performance than its compared non-HVG.

S3. The specific structure of the MNN model

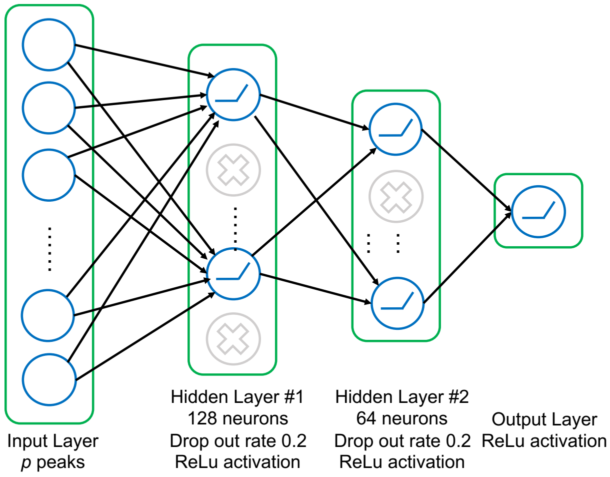

Figure S13. The specific structure of the MNN model.
